# Methotrexate Nanoparticles Prepared with Codendrimer from Polyamidoamine (PAMAM) and Oligoethylene Glycols (OEG) Dendrons: Antitumor Efficacy *in Vitro* and *in Vivo*

**DOI:** 10.1038/srep28983

**Published:** 2016-07-08

**Authors:** Yanna Zhao, Yifei Guo, Ran Li, Ting Wang, Meihua Han, Chunyan Zhu, Xiangtao Wang

**Affiliations:** 1 Institute of Medicinal Plant Development, Chinese Academy of Medical Sciences & Peking Union Medical College, No. 151, Malianwa North Road, Haidian District, Beijing 100193, China; 2 School of Pharmacy, Heilongjiang University of Chinese Medicine, No. 24, Heping Road, Xiangfang District, Harbin 150040, China

## Abstract

The novel methotrexate-loaded nanoparticles (MTX/PGD NPs) prepared with amphiphilic codendrimer PGD from polyamidoamine and oligothylene glycol dendrons were obtained via antisolvent precipitation method augmented by ultrasonication. Based on the excellent hydrophility of PGD, the drug-loaded nanoparticles could be investigated easily with the high drug-loading content (~85.2%, w/w). The MTX/PGD NPs possessed spherical morphology, nanoscaled particle size (approximately 182.4 nm), and narrow particle size distribution. Release of MTX from MTX/PGD NPs showed a sustained release manner and completed within 48 h. Hemolytic evaluation indicated MTX/PGD NPs presented good blood compatibility, and the cytotoxicity of nanoparticles against breast cancer cells *in vitro*, biodistribution in tumor tissue, and antitumor efficacy *in vivo* were enhanced significantly compared to MTX injection. According to the higher drug-loading content, enhanced antitumor efficacy, and appropriate particle size, MTX/PGD NPs as the drug delivery systems could have potential application for cancer chemotherapy in clinic.

Chemotherapy is commonly preferred for human malignancies, as surgery and radiation therapy may not be fully effective at advanced stages of cancer^1^. Methotrexate (MTX) has played an instrumental role in the treatment of various cancers^2^,^3^,^4^,^5^,^6^,^7^,^8^. However, the application of MTX is seriously limited due to its poor solubility, toxic side effects, and nonspecific drug delivery^9^. In order to improve the therapeutic efficacy and reduce the side effects, various nanoscaled drug delivery systems (NDDS) such as microemulsions^10^, liposomes^11^, nano-conjugates^12^, nanoparticles^13^, nanocapsules^14^, and polymeric micelles^15^, have been developed. Nanoparticles (NPs) are one of the typical drug delivery systems with an average particle size of nanometer range, which could improve the solubility of hydrophobic drugs, stabilize and protect drugs from degradation, facilitate targeted drug delivery, enhance accumulation in the tumor site via the enhanced permeability and retention (EPR) effect, prolong circulation time by avoiding the rapid renal clearance and reticuloendothelial systems (RES)^16^.

Based on these advantages, much research was focused on the preparation of MTX-loaded nanoparticles from linear or star amphiphilic copolymers via physical entrapment or chemical conjugation^17^,^18^,^19^,^20^,^21^. Majid Afshari *et al*. prepared MTX-loaded PLGA and PLGA-PEG nanoparticles as spherical shaped particles with a particle size of approximately 200 nm, the cytotoxicity effect was enhanced significantly^19^. Luo *et al*. developed a MTX-loaded PEG-chitosan nanoparticles system that showed enhanced cytotoxicity both *in vitro* and *in vivo*^22^. Wang *et al*. prepared core-shell nanoparticles from conjugated pullulan with MTX, these nanoparticles exhibited significant inhibitory effect on the cell proliferation^23^. Baker *et al*. researched the polyvalent dendrimer-MTX conjugate, which inhibited dihydrofolate reductase and induced cytotoxicity in KB cells^24^.

In our previous study, the amphiphilic codendrimer **PGD** from polyamidoamine (PAMAM) and oligoethylene glycols (OEG) dendrons was synthesized and utilized to prepare drug-loaded micelles (Fig. 1)^25^,^26^. Due to the highly branched structure, good biocompatibility, and environmental stimuli, **PGD** was considered to be potential feasible to be used as the drug carrier. Although these researches enhanced the solubility, promoted or maintained the cytotoxicity, the drug-loaded content (DLC) was unsatisfied (<40%), which may induce the side effect and limit its application.

Here, we report a novel strategy of preparation MTX nanoparticles (MTX/**PGD** NPs) based on amphiphilic codendrimer **PGD** via the antisolvent precipitation method augmented by ultrasonication (Fig. 1), and the DLC of MTX/**PGD** NPs was improved to 85.2% (w/w). Furthermore, the physiochemical properties of nanoparticles including the particle size, morphology, stability, release profiles, and antitumor efficacy both *in vitro* and *in vivo* were evaluated, which showed the MTX/**PGD** nanoparticles could be utilized as a potential drug delivery vehicle for breast cancer therapy.

## Results and Discussion

### Preparation of MTX/PGD NPs

**Figure 2:**
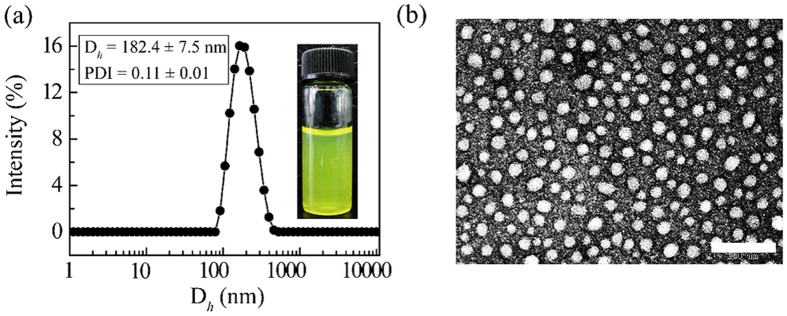
DLS curves of MTX/PGD NPs in aqueous solutions (a) and TEM images (b). Scale bar: 200 nm.

In our previous studies, the drug-loaded micelles were prepared from amphiphilic codendrimers and anticancer drugs via classic dialysis method. Here, the MTX-loaded **PGD** nanoparticles (MTX**/PGD** NPs) were prepared via the revised antisolvent precipitation method augmented by ultrasonication, the resultant nanoparticles were dispersed in deionized water with slight yellow color (Fig. 2a), and the DLC was approximately 85.2%.

### Particle Size, Zeta Potential, and Morphology of Nanoparticles

The particle size and zeta potential of MTX/**PGD** NPs in aqueous solution were measured by dynamic light scattering (DLS). The nanoparticles had a mean diameter of approximately 182.4 ± 7.5 nm and a very narrow size distribution (PDI = 0.11 ± 0.01). The particle size distribution curve is shown in Fig. 2a. The appropriate particle size (<200 nm) of MTX-loaded nanoparticles benefited to avoid reticuloendothelial system uptake and achieve passive tumor targeting through EPR effect^27^,^28^. The surface charge of this nanoparticles was approximately 15.9 ± 0.2 mV, while the surface charge of the MTX bulk powder suspensions (MTX suspension) was about −29.3 ± 1.2 mV. The reversal surface charge of the nanoparticles could be attributed to the codendrimer **PGD**, which presented positive charge and distributed on the surface of the nanoparticles.

**Figure 3:**
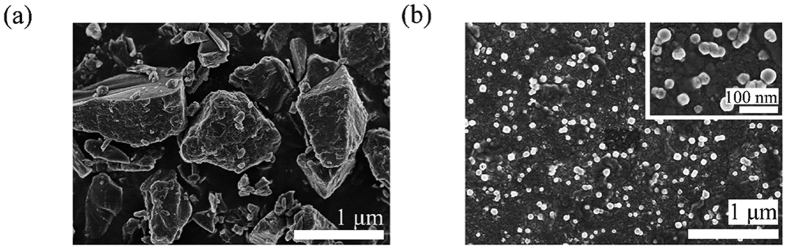
SEM images of MTX bulk powder (a) and MTX/PGD NPs (b).

Morphology of MTX/**PGD** NPs was investigated by TEM observation, the nanoparticles were well dispersed as individual particles with the regularly spherical shape, and the mean diameter was approximately 40 nm (Fig. 2b). The variation in particle size measured by TEM and particle-size analyzer was attributed to the fact that dynamic light scattering (DLS) measurement of the particle size analyzer gave the hydrodynamic diameter rather than the actual diameter of the dried particles^29^. To compare the morphology of MTX/**PGD** NPs with MTX bulk powder, they were detected by SEM further. SEM micrograph revealed that MTX/**PGD** NPs were spherical shape with approximately 50 nm in size (Fig. 3b); however, MTX bulk powder presented irregular shapes with the larger size (>1 μm, Fig. 3a), composed mostly of fragmented drug crystals. These results suggested the crystalline structure of MTX was lost during the preparation of the MTX/**PGD** NPs.

### Measurement of the Fixed Aqueous Layer Thickness

The fixed aqueous layer thickness (FALT) of MTX/**PGD** NPs was determined by the method introduced by Sadzuka and Shi^30^,^31^, which was 10.3 nm (Supplementary Information, Table S1). The FALT of MTX/**PGD** NPs was almost 10 times thicker than that of MTX suspension (approximately 1.0 nm), the result suggested that codendrimer **PGD** was distributed on the surface of these nanoparticles and branched OEG chains might form ‘brush’ structures due to the strong steric hindrance^32^. A higher FALT could possibly provide a shift of the hydrodynamic phase of shear to greater distances from the particle surface, afford steric hindrance and prevent the particles to form aggregation and/or agglomeration, hence increase the stability of the nanoparticles^2^. Accordingly, the circulation time of the nanoparticles in blood was likely to increase, and prevent the attraction of opsonins to the nanoparticles^33^.

### Surface Element Analysis

EDS analysis was employed to determine the composition and content on the surface of MTX bulk powder and MTX/**PGD** NPs (Supplementary Information, Figure S1). The amount of O element in MTX/**PGD** NPs was significantly increased compared with MTX bulk powder; meanwhile, the amount of N element in MTX/**PGD** NPs was 3-fold lower than MTX bulk powder. From these results, it seemed that the amphiphilic codendrimer **PGD** was mostly distributed on the surface of MTX/**PGD** NPs, due to the outside OEG dendron of **PGD** showed higher oxygen content, the lower N element could be attributed to the PAMAM core in **PGD**. The exposed OEG dendron chains, the same as poly(ethylene glycol) chains, could suppress the interaction between the nanoparticles and plasma proteins, decrease their immunogenicity and prolong their circulation time in the blood^34^.

### Measurement of Stability

For the storage stability study, MTX/**PGD** NPs were stored at 4 °C, 25 °C, and 37 °C, respectively. The particle sizes of these samples were monitored over 14 days by DLS without further ultrasonication (Supplementary Information, Figure S2). After 14 days, the particle sizes of MTX/**PGD** NPs were approximately 193.8 ± 7.8, 194.5 ± 8.9, and 194.7 ± 3.7 nm for 4 °C, 25 °C and 37 °C respectively, no significant difference (P > 0.05) was obtained when compared to the value at day 0 (p = 0.3, 0.3, and 0.2 for 4 °C, 25 °C, and 37 °C relatively).

The stability *in vivo* is more important in clinical application, thus the stability of MTX/**PGD** NPs in plasma at 37 °C was studied. The mean diameter of MTX/**PGD** NPs was increased from 75.6 ± 6.5 to 90.1 ± 3.8 nm throughout the stability test (Supplementary Information, Figure S2). Although the average particle size was increased slightly, no significant difference was obtained compared to day 0 (p > 0.05), indicating the high stability *in vivo*.

For the lyophilization stability study, MTX/**PGD** NPs were lyophilized directly without any protector and reconstituted with water. After reconstitution, MTX/**PGD** NPs presented the regularly spherical shape with the mean diameter of approximately 181.1 ± 9.3 nm and a narrow size distribution (PDI = 0.15 ± 0.02), the zeta potential of the reconstituted nanoparticles was 14.1 ± 0.8 mV, all of these showed no significant changes compared with MTX/**PGD** NPs before lyophilization (Supplementary Information, Figure S3), which could be explained by the peripheral hydrophilic OEG dendron hindered the aggregation of particles.

All the results suggested that the MTX/**PGD** NPs showed the enhanced stability due to the branched OEG section, the similar phenomenon had been reported broadly that PEGlation can promote the stability of nanoparticles^32^,^35^. Based on the good stability, MTX/**PGD** NPs could be researched *in vivo* further.

### Differential Scanning Calorimetry and X-ray Diffraction Analysis

To confirm the existing state of MTX in the nanoparticles, differential scanning calorimetry (DSC) was utilized to elucidate the thermal transitions of MTX in bulk, in a physical mixtures and within MTX/**PGD** NPs analyses (Supplementary Information, Figure S4). The spectrum of MTX showed a sharp endothermic peak at 148 °C, which was associated with the melting of MTX according to the reports before^36^. Thermograms of **PGD** exhibited no endotherm during the whole heating procedure. The melting endothermic peak of MTX could be detected in the physical mixtures of MTX and **PGD**, however disappeared in MTX/**PGD** NPs.

X-ray powder diffraction was also used to confirm the state of MTX in drug-loaded nanoparticles. Free MTX bulk powder, physical mixture, and MTX/**PGD** NPs were measured (Supplementary Information, Figure S4). The intense peaks indicative MTX existed as the crystalline compound, which could be observed from the spectra of MTX bulk powder and physical mixture of MTX and **PGD**. While, in the MTX/**PGD** NPs, the characteristic diffraction peaks of MTX powder disappeared, which was in good agreement with the result from DSC.

Both the results from DSC and XRD analyses verified the state of MTX in MTX/**PGD** NPs was not physical mixed simply and suggested MTX was existed as either molecular dispersion or amorphous in MTX/**PGD** NPs, the interactions between MTX and **PGD** caused these changes^37^,^38^. These results were in accordance with the results of SEM and EDS.

### *In Vitro* Studies on Release Kinetics

**Figure 4:**
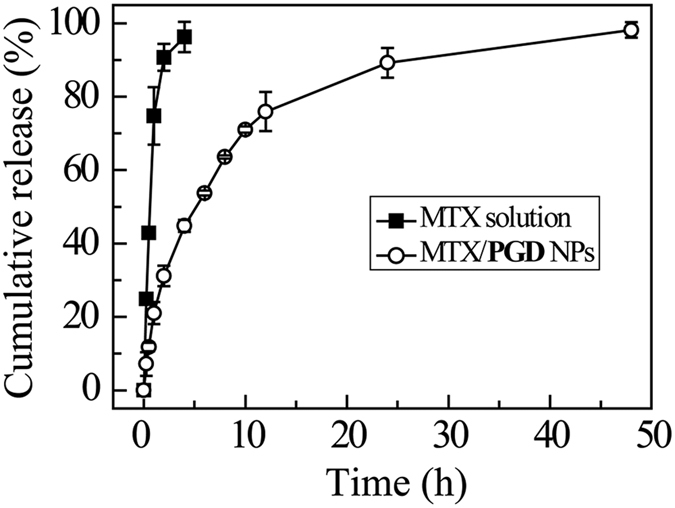
Cumulative MTX release from MTX solution and MTX/PGD NPs in 150 mM NaCl solution at 37 °C within 48 h (n = 3).

The *in vitro* drug release behavior of MTX/**PGD** NPs was evaluated in the 150 mM NaCl at 37 °C. A control experiment using free MTX was also carried out under similar conditions, and the results are shown in Fig. 4. For free MTX, complete diffusion across the dialysis membrane was found to occur within 4 h. Contrarily, the MTX/**PGD** NPs was sustained release for 48 h and the release procedure was fitted to a two-stage exponential kinetic model, with initial burst release followed by a slow separately release. Approximately 40% MTX was released from the nanoparticles within the initial 2 h, and then 60% MTX was released slowly during the following 46 h. Verified by EDS, DSC and XRD, the plausible explanation was the OEG dendrons might incorporate in the outer layer of MTX/**PGD** NPs, with the branched OEG segments extending outwards and forming a hydrophilic “shell” around the hydrophobic drug nanoparticles, which suggested that the amphiphilic codendrimer **PGD** could retain the MTX in the core of nanoparticles.

### Cytotoxicity Assay

The hemolytic activity of MTX/**PGD** NPs on rat RBC was researched to study its suitability for intravenous administration (Supplementary Information, Figure S5). After incubation with the 2% (w/v) RBC suspension at 37 °C for 5 h, the hemolysis rates of MTX/**PGD** NPs were below 2% in concentration ranging from 0.01 to 1 mg/mL (MTX equivalent concentration), suggesting no RBC membrane related toxicity.

**Figure 5:**
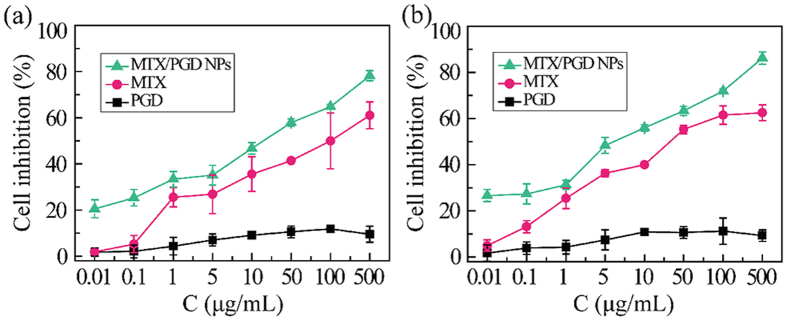
Cytotoxicities of MTX/PGD NPs towards MCF-7 cells (a) and 4T1 cells (b) after incubation for 48 h (n = 5).

To investigate *in vitro* antitumor efficacy of MTX/**PGD** NPs, the viability of two different cancer cell lines including MCF-7 cells and 4T1 cells were measured with an MTT assay, the concentration of MTX in both formulations ranged from 0.01 to 500 μg/mL. As shown in Fig. 5, amphiphilic codendrimer **PGD** showed no significant cytotoxicity against MCF-7 cells and 4T1 cells (inhibition <10%). On the contrary, MTX/**PGD** NPs inhibited the growth of MCF-7 cells and 4T1 cells in a dose-dependent manner after incubated for 48 h. MTX/**PGD** NPs exhibited enhanced cytotoxicity against MCF-7 cells, the half inhibition to cell growth (IC_50_) of the nanoparticles was detected as 8.9 μg/mL and 7.5-fold lower than MTX injection with the IC_50_ of 66.6 μg/mL (Fig. 5a). The cytotoxicity of MTX/**PGD** NPs against 4T1 cells were studied then, and the similar results were obtained as those from MCF-7 cells. MTX/**PGD** NPs exhibited a higher cytotoxicity compared to free MTX, the IC_50_ values for nanoparticles and free MTX were 3.8, 29.9 μg/mL (Fig. 5b), inhibition efficacy enhanced by 8-fold. It was generally considered that free drug was only transported into cells by passive diffusion while the nanoparticles could be nonspecifically internalized into cells via endocytosis, phagocytosis or pinocytosis after accumulating on the surface of the cells^39^. Besides, it was reported that the nanoparticles with positive charged could present better association and internalization rates due to the electrostatic interaction between the particles and the negatively charged cell membrane^39^,^40^. Hence, MTX/**PGD** NPs were potent to be much more effective in enhancing the *in vitro* inhibition effect in comparison with free MTX.

### Cellular Uptake

**Figure 6:**
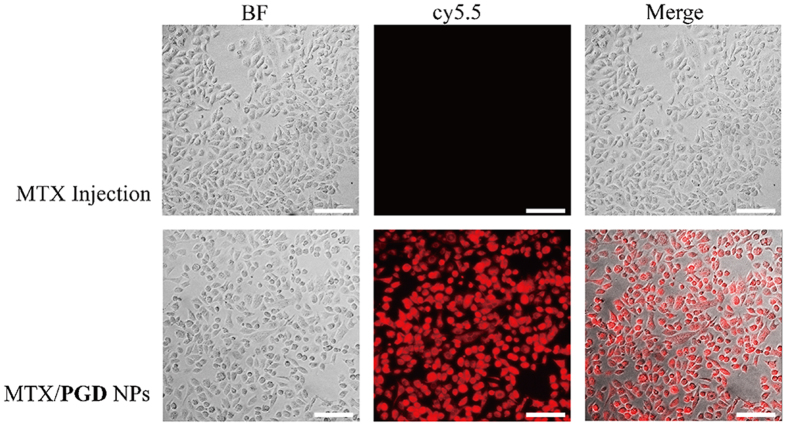
Representative fluorescent microcopy images of 4T1 cells incubated with MTX injection and MTX/PGD NPs for 4 h. Red: cy5.5, scale bar: 20 μm.

Cellular uptake of MTX/**PGD** NPs and MTX injection was characterized in 4T1 cell line using fluorescent microcopy imaging system. To qualitatively investigate the cellular uptake of the MTX/**PGD** NPs, 4T1 cells were incubated with the cy5.5-labeled agents (cy5.5 was mixed with MTX injection or encapsulated in MTX/**PGD** NPs) at the equivalent cy5.5 concentration for 4 h. The MTX injection showed weak fluorescence signals in 4T1 cells, on the contrary, MTX/**PGD** NPs exhibited greater fluorescence signals (Fig. 6). Calculated from the fluorescence intensity (185.2 ± 7.4 *vs.* 1018.5 ± 8.1, MTX injection *vs.* nanoparticles), the uptake efficacy of nanoparticles was enhanced approximately 5.5-fold (p < 0.001), indicating that the MTX/**PGD** NPs were taken up better by 4T1 cells. It was possible that nanoparticles with a diameter of approximately 180 nm could be preferentially internalized via endocytosis pathway, while free MTX was transported into cells by passive diffusion.

### *In Vivo* Antitumor Efficacy

**Figure 7:**
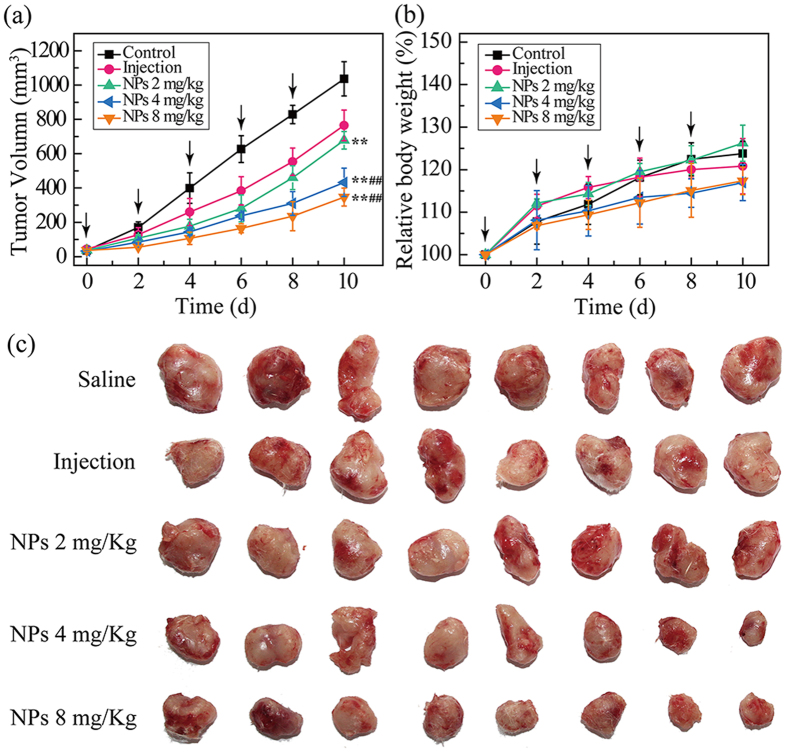
*In vivo* antitumor efficacy of MTX/PGD NPs by the intravenous route: tumor volume change of 4T1 bearing BALB/c mice (a), body weight change (b), tumor images (c) in 4T1 bearing BALB/c mice. For each animal, five consecutive doses were given (marked by arrows). Data represent mean ± SD (n = 8). ^**^p < 0.001 *vs.* saline control group, ^##^p < 0.001 *vs.* MTX injection.

Motivated by the high *in vitro* anticancer effect and cell uptake efficacy, the *in vivo* antitumor activitites of MTX/**PGD** NPs were further investigated by using BALB/c mice bearing 4T1 breast tumor model via intravenous administration, while the saline and MTX injection were used as control (Fig. 7). Tumor-bearing mice were randomly divided into five groups (n = 8): saline (blank control), MTX injection (positive control, 8 mg/Kg mice), and three MTX/**PGD** NPs agents (test groups, 2, 4, 8 mg equivalent MTX/Kg mice), which were applied every two days for 5 times. The tumor volume and body weight were monitored every two days for 10 days. At the end of this *in vivo* experiment, the tumors of all different groups were removed, photographed, and weighted. The tumor sizes from nanoparticles groups were obviously smaller than those from both blank control and MTX injection groups (Fig. 7c). As shown in Fig. 7a, the time-related tumor volume increase was observed, the tumor sizes in saline control group increased by 23.0-fold. Compared to saline control group, MTX injection group only presented moderate antitumor efficacy, tumor volume increased by 17.4-fold. In contrast, the three test groups treated with nanoparticles produced dose-dependent antitumor effects, the dose was selected as 2, 4, and 8 mg/Kg and the tumor volume increased by 18.3-fold, 13.5-fold, and 9.6-fold respectively. The statistically significant difference was obtained for mice treated with three nanoparticles groups compared with saline control group (p < 0.001), and two nanopaticles groups with the dose of 4 and 8 mg/Kg showed the statistically significant difference compared with MTX injection group (p < 0.001).

Calculated tumor inhibition rates based on the averaged weight of the tumors, which were removed from all five groups at the end of this *in vivo* experiment, further indicating the better antitumor effect by the MTX/**PGD** NPs (Supporting Information, Table S2). The average tumor weight of the saline control, MTX injection, and three test groups were 0.92 ± 0.06, 0.51 ± 0.02, 0.63 ± 0.04, 0.41 ± 0.03, and 0.20 ± 0.03 g, respectively. Relative to the saline control, the inhibition rates were 44.8% and 31.7%, 55.4%, 78.5% for the MTX injection and three nanoparticles groups, which were statistically significant different (p < 0.001). Relative to the MTX injection, the inhibition effect was enhanced by approximately 2-fold under the same dose 8 mg/Kg, furthermore, the higher inhibition effect was observed even reducing the dose to 4 mg/Kg, and both of them presented statistically significant difference (p < 0.001). Either from tumor growth progression curves or the final weight of the tumor, the MTX/**PGD** NPs exhibited higher antitumor efficacy, which was in good agreement with these results from *in vitro* antitumor efficacy and cell uptake efficacy. Enhanced inhibition activity of MTX/**PGD** NPs could be attributed to longer blood circulation, potential higher accumulation in tumor via EPR effect, and higher cell uptake efficacy^41^,^42^.

The change in the body weight of mice in the whole experiment and blood biochemistry analysis at the end of this *in vivo* experiment were carried out to investigate the adverse effects of the nanoparticles. As seen in Fig. 7b, the mice treated with all five agents grew at approximately 20% of their initial weight, and no statistically difference was observed among them (p > 0.05), suggesting a low level potential of systemic toxicity. Furthermore, liver function markers including alanine aminotransferase, aspartate aminotransferase, and the kidney function markers such as blood urea nitrogen, creatinine were measured to evaluate the safety of drug-loaded nanoparticles (Supplementary Information, Table S2). These results from test groups showed no significant difference when compared with the saline group (p > 0.05), suggesting no obvious hepatic or renal toxicity^43^. All these results of *in vivo* antitumor studies indicated that MTX/**PGD** NPs could improve antitumor efficacy without significant toxicity.

### In/*Ex Vivo* Tumor Targeted Imaging

**Figure 8:**
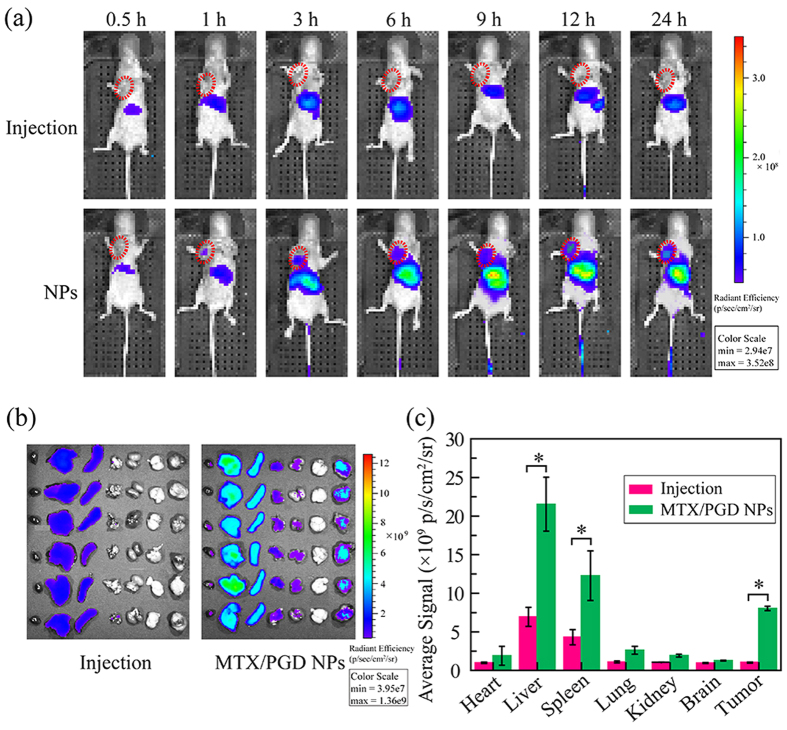
The *in vivo* and *ex-vivo* typical fluorescence images. The mice bearing 4T1 tumors were injected one time with DIR labeled MTX injection and nanoparticles (MTX equivalent, 8 mg Kg^−1^): *In vivo* images of mice after 0.5, 1, 3, 6, 9, 12, and 24 h post injection (**a**), *ex-vivo* fluorescence images of main tissues, all the organs and tumors were placed in the order of (heart, liver, spleen, lung, kidney, brain, and tumor) after 24 h post injection (**b**), intensity of fluorescent signal in organs and tumor after 24 h post injection (**c**). ^*^p < 0.01 vs. MTX injection.

To evaluate the biodistribution in various organs and tumor tissue of MTX/**PGD** NPs, DIR as the fluorescent dye was used to label nanoparticles and the fluorescence imaging study was performed with 4T1 bearing mice, the results were shown in Fig. 8. *In vivo* fluorescent images were taken at different time after intravenously. As shown in Fig. 8a, during the whole monitoring procedure, the nanoparticles were found to accumulate into tumor at 1 h after intravenous injection, while extremely weak signal was found in tumors from the mice treated with MTX injection. Meanwhile, for nanoparticle groups, strong signals were detected in livers and spleens. All mice were sacrificed at 24 h after intravenous injection, the tumors and main organs were removed and fluorescencently imaged (Fig. 8b). Compared to injection group, the tumors in nanoparticle groups showed the strong fluorescence signals (p < 0.01). Besides, the signals in liver, spleen, lung, and kidney were strengthened. The average signals further confirmed the *ex-vivo* images well as shown in Fig. 8c. These results suggested that nanoparticles were mainly distributed in the tumor tissue due to the EPR effect, as well as reticuloendothelial system (RES) organs such as liver, spleen, and kidney.

## Conclusions

In this study, amphiphilic codendrimer **PGD** was utilized to prepare spherical methotrexate nanoparticles (MTX/**PGD** NPs) via antisolvent precipitation method augmented by ultrasonication, with the mean diameter of approximately 182.4 nm and a very narrow size distribution, and the DLC was 85.2%. The FALT and surface element analysis results suggested the amphiphilic codendrimer **PGD** was dispersed on the surface of MTX nanoparticles. Besides, MTX/**PGD** nanoparticles showed higher stability and sustained release for 48 h *in vitro*. The MTX/**PGD** nanoparticles presented promising high tumor inhibition and biosafety, which was confirmed by *in vitro* cytotoxicity study, cell uptake, biodistribution in tumor tissue, *in vivo* tumor growth inhibition, and blood biochemistry analysis. These results suggested the potential advantages of MTX/**PGD** nanoparticles as safe and efficient drug delivery system for anticancer therapy in clinic.

## Materials and Methods

### Materials

Codendrimer (**PGD**) was synthesized according to pervious papers^44^. Methotrexate (MTX, purity >98%) was purchased from Shanghai Heng Yuan Bio-Tech Co., Ltd. (Shanghai, China). Dialysis membrane of molecular weight cut-off of 14000 Da was obtained from Spectrapor. Normal saline solution was purchased from Sigma Chemical Co. (America). Other reagents and solvents were purchased of analytical grade and obtained from commercial company.

The human breast cancer cell line (MCF-7 cells) and murine 4T1 breast cancer cell line (4T1 cells) were purchased from the Institute of Basic Medical Science, Chinese Academy of Medical Science (Beijing, China). RPMI-1640 was purchased from Hyclone Thermo Scientific (America). Fetal bovine serum (FBS, 10%) was purchased from Gibco Thermo Scientific (America), and 100 unit/mL penicicillin G and streptomycin were purchased from Biotopped Science & Technology Co., Ltd. (Beijing, China).

Male BALB/c mice (20 ± 2 g), male Sprague-Dawley rats (200 ± 20 g) and male nu/nu nude mice (20 ± 2 g) were purchased from Vital River Laboratory Animal Technology Co., Ltd (Beijing, China). All the animals were acclimatized in a laminar flow room at controlled temperature of 25 ± 2 °C, relative humidity of 50–60% and 12 h light-dark cycles with standard diet ad libitum for 1 week prior to experimentation. All experimental procedures comply with the Guidelines and Policies for Ethical and Regulatory for Animal Experiments as approved by the Animal Ethics Committee of Peking Union Medical College (Beijing, China).

### Preparation of MTX-PGD NPs

MTX/**PGD** nanoparticles (MTX/**PGD** NPs) were prepared via antisolvent precipitation augmented by ultrasonication. Briefly, MTX (16 mg) and **PGD** (2 mg) was dissolved in DMF (1 mL) in the glass vial, and then the DMF solution was injected into deionized water (5 mL) at room temperature under continuous ultrasonication (250 W) for 10 min. The mixed solution was transferred into the dialysis bag (MWCO 14000) and dialyzed against deionized water (4 × 1 L) for 4 h to remove DMF and the free drug. To quantify the drug-loading content (DLC), the drug in the nanoparticles was collected by lyophilized and analyzed by HPLC (UltiMate 3000, DIONEX) using a UV detector operated at 306 nm. The quantitative analysis was carried out on a Thermo C18 (4.60 mm × 250 mm, 5 μm) and compared to a calibration curve generated from 9:1 PBS (pH = 7.4, 0.1 M):CH_3_CN (y = 1.36x + 0.03, R^2^ = 0.9999). The flow rate was 1.0 mL/min, and the sample injection volume was 20 μL. The DLC was calculated as follows, the experiments were conducted in triplicates, and the data were shown as the mean values plus standard deviation (±SD).


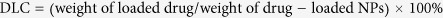


### Particle Size and Zeta Potential Measurements

The particle size, size distribution, and zeta potential analysis of MTX/**PGD** NPs were determined by the dynamic light scattering (DLS) using a Zetasizer Nano-ZS analyzer (Malvern Instruments, UK) with an integrated 4 mV He-Ne laser, λ = 633 nm, which used the backscattering detection (scattering angle θ = 173°) at room temperature. Samples were prepared in deionized water at a concentration of 1 mg/mL. The experiments were conducted in triplicates. The data were shown as the mean values plus standard deviation (±SD).

### Measurement of the Fixed Aqueous Layer Thickness (FALT)

The MTX/**PGD** NPs were centrifuged at 25 °C for 20 min at 13000 rpm to obtain the nanoparticles, and the pellet was washed with a phosphate buffer solution. Then the pellet was resuspended in NaCl solutions of different ion concentrations. The zeta potential was measured and the calculation of FALT (L) was based on the linear correlation between ln ζ (zeta potential) and κ (Debye–Huckel parameter): ln ζ = ln A−κL. Where A is regarded as constant and κ is Debye–Huckel parameter, expressed as κ = 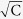/0.3 for univalent salts where C is the molarity of electrolytes. The slope L gives the position of the slipping plane or thickness of the fixed aqueous layer in nm units. The experiments were conducted in triplicates. The data were shown as the mean values plus standard deviation (±SD).

### Transmission Electron Microscope

Transmission electronic microscopy (TEM) measurements were performed on a JEM-1400, operating at an acceleration voltage of 80 kV. A drop of MTX/**PGD** NPs (0.2 mg/mL) was placed on carbon-coated copper grid. After 2 min, the grid was drained by filter paper to remove the aqueous solution, air-drying at room temperature, and then dyeing with uranyl acetate solution (2%, w/v).

### Scanning Electron Microscope and Surface Element Analysis

The morphology and surface chemical composition of MTX bulk powders and MTX/**PGD** NPs were investigated by scanning electron microscopy (SEM) with Energy Dispersive Spectrometer (SEM-EDS; S-4800, Hitachi Limited., Tokyo, Japan). MTX bulk powders and MTX/**PGD** NPs were lyophilized directly, a drop of MTX/**PGD** NPs solution (0.2 mg/mL) was placed on matrix and air-drying, then these samples were sputter-coated with a conductive layer of gold-palladium (Au/Pd) for 1 min. An accelerating potential of 30 mV was used for the observation and analysis.

### Stability Study

For the storage stability study, MTX/**PGD** NPs were stored at 4, 25, and 37 °C over 14 days separately. The particle size and polydispersity index (PDI) of the samples were measured at 0, 1, 3, 7, and 14 days. The experiments were conducted in triplicates, and the data were shown as the mean values plus standard deviation (±SD).

For the stability study under physiological conditions, MTX/**PGD** NPs were suspended in plasma at 37 °C over 12 h, and the particle size of the samples was measured at 0, 1, 3, 6, 9, 12 h. The experiments were conducted in triplicates, and the data were shown as the mean values plus standard deviation (±SD).

For the lyophilized stability study, after freeze-drying, MTX/**PGD** NPs were resuspended in water under transient ultrasonication to their original concentration and characterized by DLS. The particle size, PDI, and zeta potential were measured. The experiments were conducted in triplicates, and the data were shown as the mean values plus standard deviation (±SD).

### Differential Scanning Calorimetry

Differential scanning calorimetry (DSC) measurements were carried out in aluminum pans with lids using a DSC Q200 (TA Co., USA) under dynamic nitrogen atmosphere. Thermograms were obtained by heating the samples from 20 to 280 °C with a scan rate of 10 °C/min.

### X-ray Diffraction Analysis

The X-ray diffraction (XRD) analysis was performed using graphite filtered CuKα radiation (λ = 1.54 Å) at 40 KV and 100 mA with a scanning rate of 8 degree per minute (2θ from 3° to 80°) by a Japan D/Max 2500PC X-ray diffractomer (Rigaku, Japan) at room temperature. MTX mixed with **PGD** in the same ratio of MTX/**PGD** nanoparticles were used for all the experiments.

### *In vitro* Studies on Release Kinetics

*In vitro* release characteristics of MTX/**PGD** NPs were studied by the dialysis method. Briefly, MTX/**PGD** nanoparticles solution (1 mL) was placed in a dialysis bag (MWCO 14000), then immersed in 50 mL of 150 mM NaCl solution. The release studies were performed at 37 °C with continuous magnetic stirring at 100 rpm under sink conditions. A control experiment using of MTX injection solution was also carried out under similar conditions. At predetermined time intervals, 5 mL external solution was withdrawn for analysis and an equal volume of fresh media was replenished. The drug release study was performed for 48 h and all release experiments were performed in triplicates. The amount of MTX released was quantified by a UV-HPLC, the data were shown as the mean values plus standard deviation (±SD).

### Hemolytic Effect

Approximately 2 mL of blood was taken from the orbital venous plexus of a male Sprague–Dawley rat and centrifuged at 3000 rpm for 5 min. The plasma supernatant was removed, and the erythrocytes were resuspended in normal saline solution. The MTX/**PGD** NPs were incubated with the 2% (w/v) RBC suspension at 37 °C for 5 h with different concentrations. Then the RBCs were removed by centrifugation, 150 μL of the supernatant was pipetted into a 96-microwell plate, and the absorbance was measured at 540 nm using a microplate reader (Versamax Tunable Microplate Reader). The results were expressed as percentage hemolysis with the assumption that deionized water caused 100% hemolysis and NS solution 0% hemolysis. The experiments were conducted in triplicate, and the data were shown as the mean values plus standard deviation (±SD).

### MTT Assays

The *in vitro* cytotoxicity against the human breast cancer cell line (MCF-7 cells) and murine 4T1 breast cancer cell line (4T1 cells) were evaluated by an MTT assay. Briefly, cells were seeded in RPMI-1640 medium supplemented with 10% fetal calf serum, and 100 units/mL penicicillin G and streptomycin at 37 °C with 5% CO_2_ in 96-well plates at a density of 8000 cells per well. After incubation for 48 h, the growth medium was replaced with fresh RPMI-1640. Then, **PGD**, MTX/**PGD** NPs, and MTX injection were added into the wells. After incubation for 48 h, 10 μL MTT solutions (5 mg/mL) were added to each well and incubation were continued for another 4 h. The medium was removed and 150 μL DMSO was added into each well to dissolve the formazan by pipetting up and down for several times. The absorbance of solution in each well was measured using an ELISA plate reader at a test wavelength of 570 nm to determine the OD value. The cell inhibition rate was calculated as follows. Cell inhibition = (1-OD_treated_/OD_control_) × 100%, where OD_treated_ was obtained for the cells treated by the nanoparticles, OD_control_ was obtained for the cells treated by the culture medium, and the other culture conditions were the same. Each experiment was done in quintuplicates. The data were shown as the mean values plus standard deviation (±SD).

### *In Vitro* Cellular Uptake of MTX/PGD NPs

The 4T1 cells (1 × 10^5^ per cell) were seeded in a 6-well plate and cultured at 37 °C for 24 h in a humidified atmosphere with 5% CO_2_, then the cy5.5 labeled agents were added into the 6-well plate. After 4 h of additional incubation at 37 °C, the medium was removed and the cells were washed with PBS three times, fixed with 4% of paraformaldehyde PBS solution for 15 min. The cellular uptake images were recorded with Delta Vision Microscopy Imaging Systems (λ = 670 nm), and all the images and average fluorescence intensity were recorded under the same condition.

### *In Vivo* Antitumor Activity

*In vivo* antitumor activity was evaluated using 4T1 bearing BALB/c mice models. Briefly, male BALB/c mice (5–6 weeks, 18–22 g) were induced 4T1 tumor by subcutaneous injection of 0.2 mL cell suspension containing 2 × 10^6^ 4T1 cells into the right armpit. When tumor exceeded 50 mm^3^ (5 days after implantation), mice were randomly divided into 5 groups (8 mice per group). Mice were administrated with saline (control group), MTX injection at a dose of 8 mg/Kg (positive group), MTX/**PGD** NPs at a dose of 2, 4, and 8 mg/Kg (test groups) in the final volume of 0.2 mL via the tail vein every 2 days for 5 times. Tumor volume was measured every two days with a caliper, and calculated by the following formula: tumor volume (mm^3^) = 0.5 × L × W^2^, where L and W represent the largest diameter and the smallest diameter, respectively. The body weight of the mice was monitored as an index of systemic toxicity. All animals were sacrificed on the 10^th^ day and the tumors were excised, weighted. The inhibition rate of the tumor was calculated as follows: IR = (tumor weight of positive or treated group/tumor weight of the control group) × 100%. The data were shown as the mean values plus standard deviation (n = 8).

Plasma samples separated from the blood samples were also collected at the end of the experiment by centrifugation. The level of aspartate aminotransferase (AST), alanine aminotransferase (ALT), blood urea nitrogen (BUN) and creatinine (CRE) were determined by routine clinical laboratory techniques.

### Biodistribution in 4T1-Tumor Bearing Mice

To determine the *in vivo* biodistribution further, male nu/nu nude mice bearing 4T1 tumor model was established. Briefly, 2 × 10^6^ 4T1 cells were inoculated subcutaneously into the right armpit of the nude mice (4–5 weeks, 14–16 g). When the tumor exceeded 200 mm^3^ (10 days after implantation), the mice were randomly divided into 2 groups (6 mice per group), and treated with DIR/MTX solution and DIR/MTX NPs intravenously at the dosage of 0.2 μg DIR/mice, respectively. The real-time distribution and tumor accumulation of free DIR/HCPT solution and DIR/HCPT NPs were recorded at 0.5, 1, 3, 6, 9, 12, and 24 h post injection using an *in vivo* imaging system (IVIS Spectrum 200, Perkin-Elmer Co., MA, USA). Mice were sacrificed at 24 h after post-injection and the tissues were excised and observed by the imaging system. The signal intensity of different tissues was quantified as the sum of all the detected photon counts within the region of interest (ROI) in the unit of [p/s/cm^2^/sr]/[μW/cm^2^].

### Statistical Analysis

The results obtained from hydrodynamic diameter, drug-loading and release experiments, cytotoxicity were expressed as the mean ± standard deviation (SD). Statistical analysis was performed with SPSS 19.0 software. Student’s t-test was used to evaluate the differences between groups, and P < 0.05 was considered significant.

## Additional Information

**How to cite this article**: Zhao, Y. *et al*. Methotrexate Nanoparticles Prepared with Codendrimer from Polyamidoamine (PAMAM) and Oligoethylene Glycols (OEG) Dendrons: Antitumor Efficacy *in Vitro* and *in Vivo*. *Sci. Rep.* **6**, 28983; doi: 10.1038/srep28983 (2016).

## Supplementary Material

Supplementary Information

## Figures and Tables

**Figure 1:**
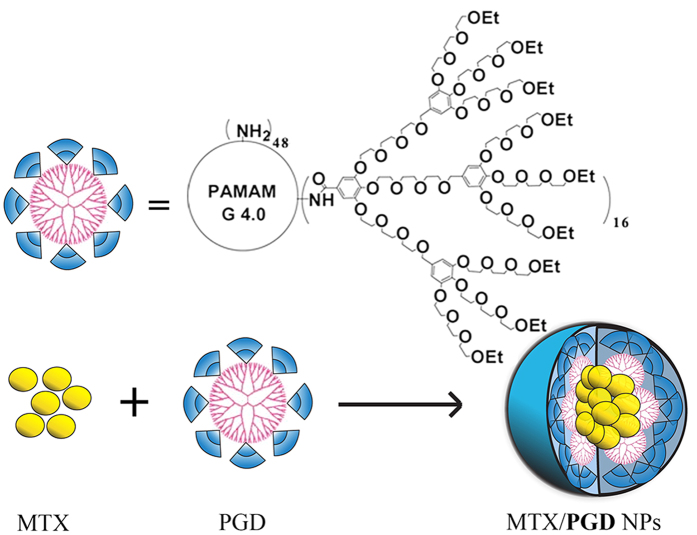
Structure of the codendrimer PGD and MTX/PGD NPs.
