# Supplementary material for: Methotrexate Nanoparticles Prepared with Codendrimer from Polyamidoamine (PAMAM) and Oligoethylene Glycols (OEG) Dendrons: Antitumor Efficacy in Vitro and in Vivo
Source: Sci Rep. 2016 Jul 8;6:28983. doi: 10.1038/srep28983 (PMC4937365; doi:10.1038/srep28983)
Supplement: Supplementary Information [file srep28983-s1.docx]

Supplementary Information

**Methotrexate Nanoparticles Prepared with Codendrimer from Polyamidoamine (PAMAM) and Oligoethylene Glycols (OEG) Dendrons: Antitumor Efficacy *in Vitro* and *in Vivo***

Yanna Zhao,^a^ Yifei Guo,^a,*^ Ran Li,^b^ Ting Wang,^b^ Meihua Han,^a^ Chunyan Zhu,^a^ Xiangtao Wang ^a,*^

^a^ Institute of Medicinal Plant Development, Chinese Academy of Medical Sciences & Peking Union Medical College, No. 151, Malianwa North Road, Haidian District, Beijing 100193, China

^b^ School of Pharmacy, Heilongjiang University of Chinese Medicine, No. 24, Heping Road, Xiangfang District, Harbin 150040, China

^*^ Corresponding author. E-mail: [ffguo@163.com](mailto:ffguo@163.com).

**Table S1.** Fixed aqueous layer thickness (FALT) of MTX suspension and MTX/**PGD** NPs^[a]^

|  | Zeta potential of NaCl concentration (mMol/L)/mV | | | | | FALT  (nm) |
| --- | --- | --- | --- | --- | --- | --- |
|  | 0 | 2 | 3 | 4 | 5 |  |
| suspension | -29.3 ± 1.2 | -27.2 ± 0.3 | -25.8 ± 0.9 | -24.9 ± 0.8 | -24.0 ± 0.1 | 1.0 |
| Nanoparticles | 15.9 ± 0.2 | 9.5 ± 0.1 | 5.2 ± 0.1 | 4.8 ± 0.1 | 3.9 ± 0.3 | 10.3 |

^[a]^ Detected by dynamic light scanning, 1 mg/mL, n = 3.

**
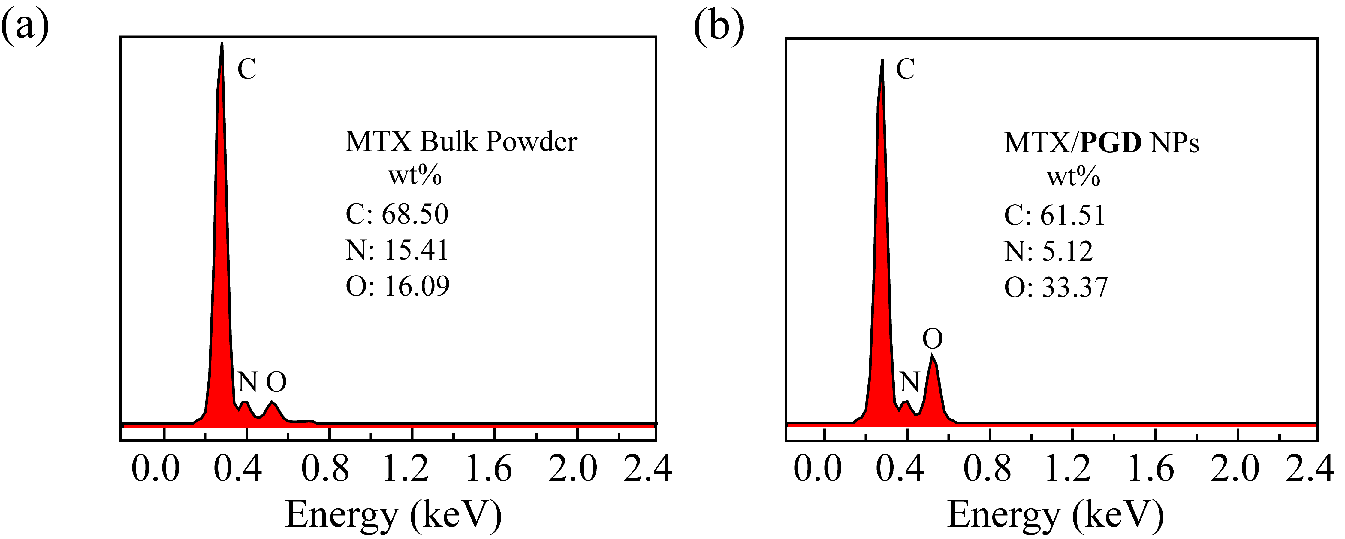
**

**Figure S1.** The major element percentage on the surface of MTX bulk powder (a) and MTX/**PGD** NPs (b).


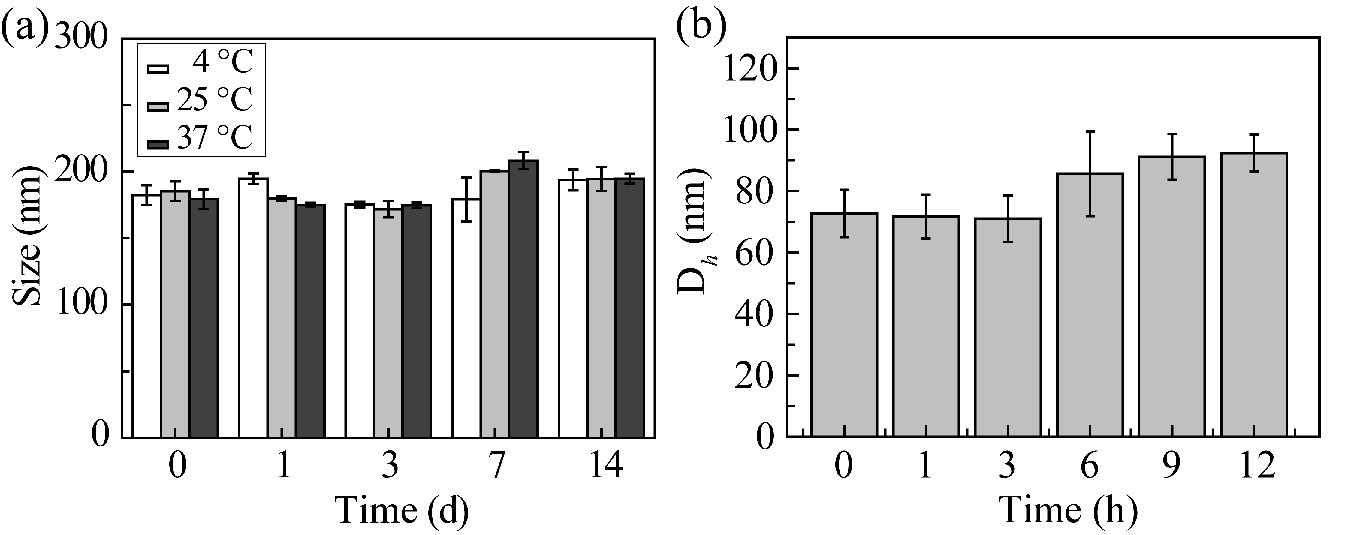


**Figure S2.** Storage stability study of MTX/**PGD** NPs at different temperature (a), plasma stability of MTX/**PGD** NPs at 37 °C (b)**.**


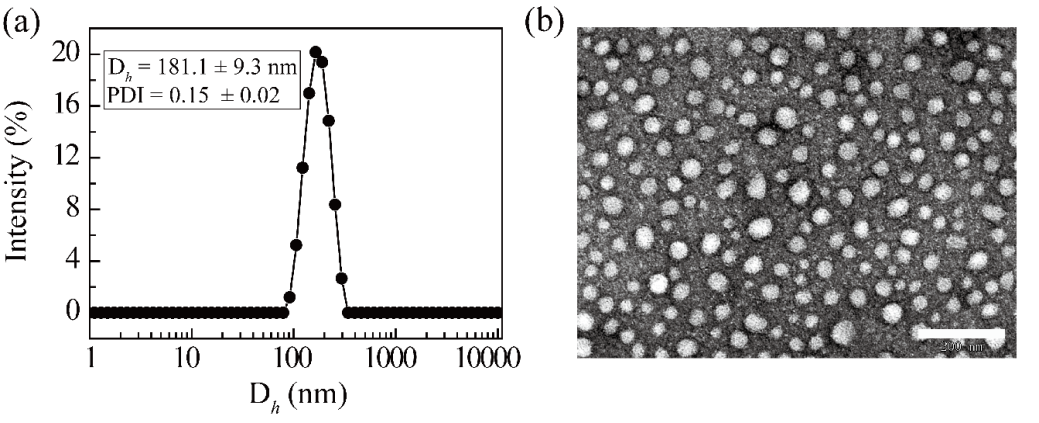


**Figure S3.** DLS curves (a) and TEM image (b) of reconstituted MTX/**PGD** NPs after lyophilization. Scale bar: 200 nm.


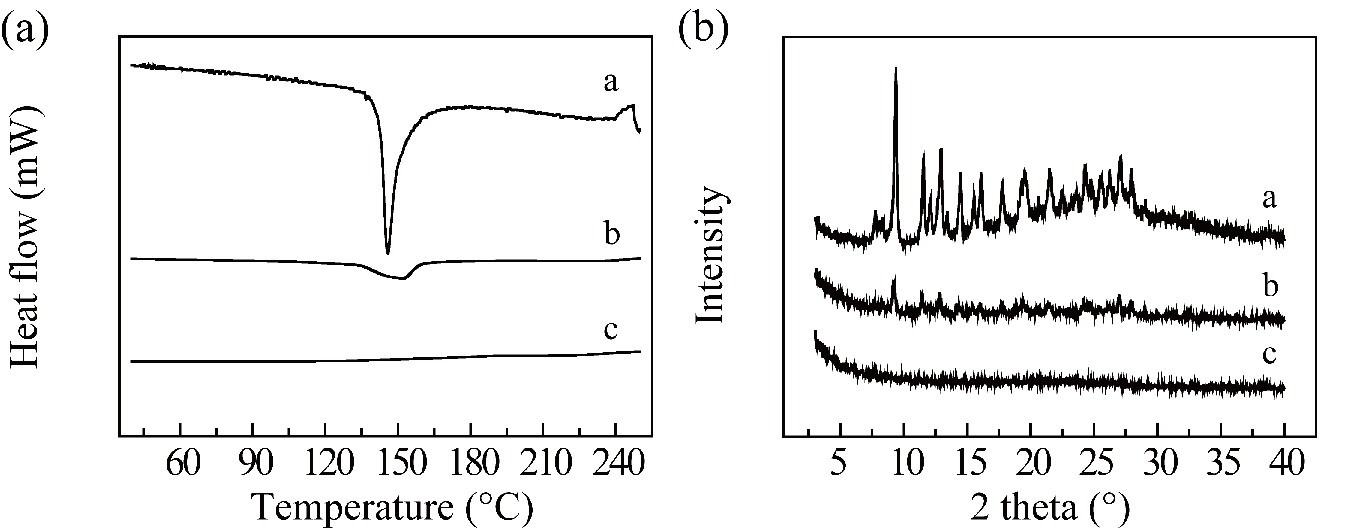


**Figure S4.** DSC thermograms (a) and XRD patterns (b) of free MTX (a), the physical mixture of MTX and **PGD** (b), and MTX/**PGD** NPs (c).


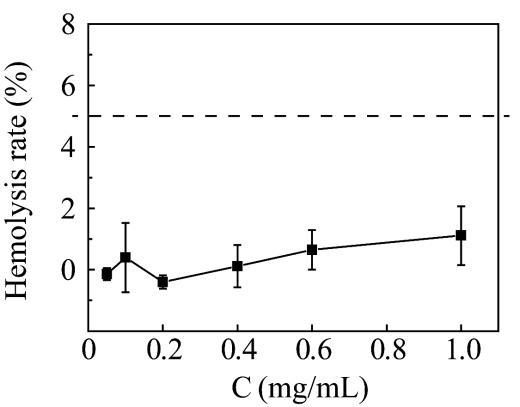


**Figure S5**. Hemolytic activity of MTX/**PGD** NPs with different concentration (n = 3).

**Table S2.** Antitumor efficacy and plasma biochemical levels of 4T1 bearing BALB/c mice ^[a]^

| Group | Dose  (mg/kg) | TW^[b]^  (g) | IR^[c]^  (%) | ALT^[d]^  (IU/L) | AST^[e]^  (IU/L) | BUN^[f]^  (mmol/L) | CRE^[g]^  (μmol/L) |
| --- | --- | --- | --- | --- | --- | --- | --- |
| Saline | — | 0.92 ± 0.06 | — | 31.5 ± 3.0 | 139.8 ± 3.4 | 5.4 ± 0.3 | 15.4 ± 0.4 |
| Injection | 8.0 | 0.51 ± 0.02 | 44.8 | 33.3 ± 4.3 | 135.2 ± 14.0 | 5.4 ± 0.2 | 16.7 ± 1.7 |
| NPs | 2.0 | 0.63 ± 0.04 | 31.7 | 31.6 ± 2.7 | 137.2 ± 13.8 | 5.1 ± 0.2 | 18.2 ± 0.1 |
|  | 4.0 | 0.41 ± 0.03 | 55.4 | 31.2 ± 1.1 | 136.6 ± 16.0 | 5.7 ± 0.2 | 18.2 ± 2.9 |
|  | 8.0 | 0.20 ± 0.03 | 78.5 | 31.8 ± 3.4 | 138.2 ± 9.9 | 4.9 ± 0.3 | 16.1 ± 2.6 |

^[a]^ mean ± SD (n = 8). ^[b]^ Tumor weight. ^[c]^ Inhibition rate. ^[d]^ Alanine transaminase. ^[e]^ Aspartate transaminase. ^[f]^ Blood urea nitrogen. ^[g]^ Creatinine.
